# Unusual primary breast cancer – malignant peripheral nerve sheath tumor: a case report and review of the literature

**DOI:** 10.1186/s13256-017-1332-1

**Published:** 2017-06-17

**Authors:** Md Shuayb, Rabeya Begum

**Affiliations:** 1 Oncology & Radiotherapy Centre, Square Hospitals Ltd, Dhaka-1205, Bangladesh; 2 USAID DFID NGO Health Services Delivery Project, Population Service & Training Centre (PSTC), House 93/3, Road 8, Block C, Niketon, Gulshan 1, Dhaka-1212, Bangladesh

**Keywords:** Malignant peripheral nerve sheath tumor, MPNST, Rare tumor, Breast cancer

## Abstract

**Background:**

Sarcomas are a rare type of breast malignancies and malignant peripheral nerve sheath tumors of the breast are even rarer. There are no specific clinical and radiological features for the diagnosis of this tumor and histological features are also reported to be nonspecific. Therefore, immunohistochemistry is required for its diagnosis. A definitive treatment protocol is unavailable because of its rarity.

**Case presentation:**

We report a case of a sporadic form of breast malignant peripheral nerve sheath tumor found in a 16-year-old Asian Bangladeshi girl. She experienced local recurrence and she had multiple left breast lumps four times in a very short period after repeated surgeries. However, she was later managed successfully with chemotherapy and locoregional radiotherapy. A chemotherapy protocol with ifosfamide, vincristine, and actinomycin was used and radiotherapy was given with a total dose of 50 Gy given in 25 fractions of 2 Gy by a 6 MV photon linear accelerator followed by 10 Gy boost given in 5 fractions of 2 Gy by 9 MeV electron energy. With more than 3 years of periodic follow-up, she is still well without any locoregional and metastatic recurrence.

**Conclusions:**

This report suggests proper immunohistochemical analysis whenever a breast sarcoma is found in order to find a rare histological variety. We believe that malignant peripheral nerve sheath tumor of the breast can be managed by total mastectomy followed by adjuvant chemotherapy and radiotherapy. Long-term meticulous follow-up is required to develop an optimum therapeutic strategy.

## Background

Rare tumors have always been intriguing curiosities for physicians. Breast cancer is the most common cancer of women in the world [1], but breast cancer of mesenchymal origin is rare. While the most common histological subtype of primary breast neoplasm remains epithelial-derived invasive ductal adenocarcinoma, comprising 75% [2], sarcomas are very infrequent accounting for less than 1% of all primary breast neoplasms [3, 4].

Malignant peripheral nerve sheath tumors (MPNSTs) are a rare type of malignancy and represent only 5 to 10% of all malignant soft tissue sarcomas [5]. Their incidence is 1:100,000 [6]. They are mostly associated with von Recklinghausen’s neurofibromatosis (VRN), with an incidence of 4%, but also occur in the general population in 0.001% of cases [7]. The common sites of involvement in decreasing frequencies are trunk (51%), extremities (45%), and head and neck (4%) [8]. MPNST of breast origin is a very rare occurrence [5].

Unfortunately, MPNST has similarities with other primary breast sarcomas which can make diagnosis difficult [9, 10]. MPNST of the breast is often unsuspected and may be misdiagnosed without high clinical suspicion and immunohistochemistry (IHC) [5]. In our review of the literature, only 15 cases have been reported from 1983 to the present [5, 9–22]. Here we report the case of a patient who presented with recurrent multiple MPNST of the breast, treated with surgical excision followed by chemotherapy and radiation therapy with more than 3-year follow-up.

## Case presentation

A 16-year-old Asian Bangladeshi girl presented to our oncology department with a 1-year history of recurrent multiple left breast lumps. She had experienced menarche at the age of 13 and did not use oral contraceptives. She had no family history of breast cancer, did not present any features of neurofibromatosis, and she had no history of prior chest irradiation.

Before coming to us, she underwent surgery four times over the last year. On her first presentation, she presented with five small painless lumps in the upper inner quadrant of her left breast. Her complete blood count, liver function test, serum electrolytes, and creatinine were all within normal limits. The tumor marker CA15-3 was 21 U/ml. We performed an excision and biopsy. The excised surgical specimen consisted of nodular pieces of tissue, the larger one of which measured 11×11×6 cm and the smaller one was 4×3×2 cm. The cut surface of the larger one was mostly solid with cleft-like spaces. Other areas were solid, slimy, and lobulated. The cut surface of the smaller piece was gray brown and slimy. On microscopic examination, the larger breast tissue showed features of a phylloides tumor. It was composed of benign glandular elements supported by cellular stromal components. Most of the stromal cells were uniform in appearance. Some of these showed mild nuclear atypia. Mitotic figures were present in small number. Focal areas showed infarction. A section from the smaller nodule revealed features of fibroadenoma.

Following surgical excision, she was well for approximately 4 months but then developed multiple painless nodular lesions at the same breast. Ultrasound of her left breast identified five hypoechoic lesions (3.2×2.6 cm, 1.5×1.2 cm, 0.9×0.8 cm, 0.8×0.6 cm, 1.7×1.1 cm) at the upper inner quadrant and one lesion (1.9×1.5 cm) at the lower inner quadrant. A 2.8 cm lymph node was noted in her left axilla. Important blood chemistries and tumor marker CA15-3 were normal. Subsequently, an excision and biopsy were carried out. The excised specimen consisted of four irregular pieces of tissue, the largest one measuring 4×3×3 cm. The cut surface was gray brown, mucinous, and slimy. Its microscopic appearance showed a stromal tumor composed of spindle cells. These were arranged in sheets of interlacing bundles. A mild degree of pleomorphism was seen. Mitotic figures were present at the rate of 0 to 1 per high-power field (HPF). A diagnosis of phylloides tumor was made.

She then had multiple nodular painful lesions along with fever 15 days later. Fine needle aspiration was performed, the cytology of which was suggestive of malignant phylloides tumor. A smear showed highly cellular material composed of spindle-shaped cells arranged in clusters and singly. Many of the cells revealed large hyperchromatic nuclei. Her complete blood count showed neutrophilic leukocytosis and a chest radiograph showed pneumonitis in her left lung. A modified radical mastectomy was done after a week. On gross examination, the specimen was attached to skin and nipple measuring 15×12×17 cm in diameter. The cut surface showed a growth measuring 10 cm in diameter. It grossly involved the deep margin. On gross examination, three lymph nodes were found of which the largest was 1 cm and the smallest 0.8 cm in diameter. A histopathologic examination revealed a malignant tumor composed of anaplastic oval to spindle cells arranged in fascicles and sheets and in herringbone pattern. Mitosis was seen >10/10 HPF. The deep surface was involved by the tumor. The underlying skin was free of tumor. The three lymph nodes showed reactive changes. It was concluded to be a malignant mesenchymal tumor, compatible with low grade fibrosarcoma. This biopsy specimen was reviewed which described a malignant tumor made of spindle-shaped cells arranged in an interlacing pattern. Mitotic figures were frequent. Areas of tumor necrosis were seen. No ductal component was found. It was diagnosed as stromal sarcoma.

She, however, developed multiple nodular lesions along the scar mark of her left chest very soon: 2 weeks later. Her thoracic radiograph and abdominal ultrasound were unremarkable. Laboratory results such as full blood count and liver enzymes including tumor marker CA15-3 were normal. Approximately 2 months later she underwent wide local excision of the tumors. On macroscopic examination, the specimen consisted of a resected partially skin-covered left-sided breast tissue measuring approximately 11×9×4 cm. The surface showed a scar mark measuring approximately 9 cm in length. The cut surface showed solid gray-white fleshy growth measuring approximately 5×3 cm. Skin and deep resection margin were grossly involved. On microscopic examination, sections made from the surgical specimen showed a malignant tumor composed of predominantly atypical spindle cells with variable cellularity and moderate to marked pleomorphism. Some of these sections showed alternating hypercellular and hypocellular areas of tumor with palisading spindle-shaped cells and intervening myxoid areas. The number of mitosis was 2 to 3/10 HPF. However, no ductal component was seen. The tumor was 0.3 cm away from the skin. The deep resection margin was involved by the tumor. Superior, inferior, medial, and lateral margins were free from the tumor. A provisional diagnosis of high grade stromal sarcoma with a differential diagnosis of malignant peripheral nerve sheath tumor was made. Immunohistochemistry was done to categorize the tumor. The tumor cells were weakly positive for S100 but negative for CD10. Therefore, a final diagnosis of MPNST was made immunohistochemically.

She subsequently developed discharge from the scar area 10 days after surgery. Among the important blood chemistries, a complete blood count showed anemia with hemoglobin (Hg) level of 8.1 g/dl and neutrophilic leukocytosis with a white blood cell count of 13,700/mm^3^ and neutrophil count of 85.2%. Serum tumor marker CA15-3 was 24 U/ml. One month after surgery, when her blood counts were normal, chemotherapy with injection of vincristine (2 mg) on day 1, injection of actinomycin (2 mg) on day 1, and injection of ifosfamide (4000 mg) on days 1 and 2 with injection of mesna coverage was started. A total of six cycles were given at 3 weekly intervals. After completion of chemotherapy, she was reviewed with no lump in either of her breasts, no discharge, and no adenopathy. Two weeks after chemotherapy, a mammogram of her right breast was done which was unremarkable. Ultrasound of her right breast was also normal. Then she was referred for locoregional radiation therapy. She received three-dimensional conformal radiation therapy with total dose of 50 Gy in 25 fractions using 6 MV linear accelerator followed by 10 Gy in five fractions of boost to tumor bed using 9 MeV electron energy. After this treatment, she was reviewed periodically at intervals of 3 months for the first 2 years and then at intervals of 4 months. To date, she is asymptomatic and her imaging is negative for local, regional, and metastatic recurrence. Her isotope whole body bone scan, chest radiograph, abdominal ultrasound, and tumor marker CA15-3 have all been unremarkable for more than 3 years.

## Discussion

MPNST is the term for tumors originating from peripheral nerves or their sheaths [23]. Cells associated with the nerve sheath include Schwann cells and perineural cells. In other contexts, its diagnosis can only be made in the presence of one of the following features: (1) the tumor arises from a peripheral nerve; (2) the tumor arises from a preexisting benign or other MPNST; (3) the tumor shows histological features of Schwann or perineurial cell differentiation [23, 24]. MPNST may arise from a precursor plexiform neurofibroma, a benign tumor characterized by differentiated Schwann cells embedded in a varied microenvironment consisting of perineural-like cells, vascular cells, fibroblasts, and mast cells [23]. In contrast, schwannomas, which are benign peripheral nerve sheath tumors composed exclusively of Schwann cells, do not give rise to malignancies [23]. MPNSTs do not include tumors belonging to the epineurium or the vasculature of peripheral nerves [23]. MPNST has replaced previous less well-defined entities of malignant schwannoma, malignant neurilemmoma, neurogenic sarcoma, and neurofibrosarcoma [22, 25].

MPNSTs comprise approximately 5 to 10% of all malignant soft tissue sarcomas, which are a very small fraction of a group of cancers that affect 1 in 100,000 people per year [5, 6]. Whereas MPNST may arise at any age with no gender predilection, its prevalence is earlier than other sarcomas, which tend to occur in the sixth decade of life [23]. The mean age of its diagnosis is 30 years but patients with neurofibromatosis type 1 (NF1), also known as VRN, are diagnosed 10 years earlier [6]. The reported case here in this article is a girl aged 16. Thus age predisposition of this case is coexisting.

Half of MPNSTs arise in the general population, and the other half derive from nerves involved by neurofibroma as part of NF1. NF1 is an autosomal dominant condition that represents the most common human cancer genetic predisposition syndrome, affecting 1 in 3000 live births [23]. NF1 is characterized by multiple areas of cutaneous hyperpigmentation, known as café-au-lait spots, and numerous neurofibromas, which are the slowly progressing, pathologically heterogeneous nerve sheath tumors [23]. Other clinical features of NF1 include axillary freckling, optic gliomas, iris hamartomas termed Lisch nodules, bone dysplasia, and family history of NF1 in a first degree relative; NF1 is diagnosed in the presence of any two of these seven criteria. Other pathologies such as cardiovascular abnormalities, learning deficiencies, and a variety of malignancies such as rhabdomyosarcoma, leukemia, and gastrointestinal stromal tumor can also be associated with NF1 [23]. Among the other half of MPNSTs that occur in the general population, approximately 40% are of sporadic form, and the remaining 10% arise secondary to previous irradiation [23, 26]. MPNST may develop within the irradiated field after a long latent period of 9 to 36 years [27], and account for 5% of radiotherapy-induced sarcoma [23]. Radiotherapy-induced MPNSTs have been observed with inferior outcome compared with sporadic or NF1-associated MPNST [28, 29]. Our patient had no history of chest irradiation and did not clinically manifest any of the features of NF1 mentioned above; hence, her case is under the pathogenesis of sporadic MPNST. Demonstration of the underlying molecular mechanisms of this type of MPNST is beyond the scope of this article.

Most MPNSTs are derived from major nerve trunks, associated with the sciatic nerve, brachial plexus, and sacral plexus [21]. Consequently, the trunk and the proximal portions of the upper and lower extremities are the most common anatomical sites [22]. A comparatively small number of MPNSTs arise in the head and neck; usually, they involve the large cranial nerves [22]. Past reviews of the literatures indicated that MPNSTs can occur almost anywhere, even in the retroperitoneum, but a more peripheral location on the extremities is comparatively common in the solitary form, whereas a central appearance on the trunk or head and neck prevails in neurofibromatosis [22]. The breast is an extremely rare location for MPNST; in the scope of our search we found descriptions of only 15 cases in the literature [5, 9–22]. Thus we report probably the 16th case of MPNST as a primary breast cancer.

Clinical diagnosis of MPNST of the breast is very difficult because of its rarity and the absence of particular symptoms or signs apart from a palpable breast lump [5, 20]. Patients with MPNST, in most cases, present with a mass sized greater than 5 cm [23, 25] and up to 50% manifest metastatic disease at presentation, usually to the lung [23]. Because of its size and consistency, it can also be confused with cystosarcoma phylloides, sarcomatoid carcinoma, and carcinosarcoma of the breast [18, 20]. The background setting of NF1 should hint of the possibility of MPNST but few MPNSTs of the breast have appeared with genetic predisposition of NF1: 4 out of 15 cases, as reported so far, to the best of our knowledge (Table 1) [5, 9–22]. Thus clinical suspicion of sporadic MPNST of the breast remains challenging. In our case, there were multiple lumps in our patient’s breast (the largest lump measuring 11×11 cm), and metastases were not evident. It was of the sporadic type and could not be assumed clinically.

**Table 1:** Published case reports on malignant peripheral nerve sheath tumor of the breast

Author	Ethnicity	Age in years	Sex	Genetic/Sporadic	Tumor size	Axillary LN involvement	Treatment received	Follow-up (months)	Local recurrence	Distant metastases
Catania *et al*. (1992) [11]	Italian	(Further information from this article could not be accessed in the scope of our search)								
Hauser *et al*. (1995) [14]	Austrian	27	F	Sporadic	1.2 cm	No	Wide local excision followed by RT (50 Gy over 5 weeks) followed by chemotherapy with doxorubicin, ifosfamide, and dacarbazine	18	No	Sarcomatous pleuritis (10 months after surgery)
Malas *et al*. (1995) [12]	South African	71	F	NF1	6 cm	No	Mastectomy with axillary clearance followed by RT (40 Gy in 10 alternate day fractions)	24	No	No
Berrada *et al*. (1998) [15]	French	26	F	Sporadic	8 cm	No	Mastectomy with axillary clearance	16	Yes	Lung and bones (after 11 months)
Medina-Franco *et al*. (2003) [13]	Mexican	4	F	NF1	2.5 cm	No	Wide local excision followed by RT	No info	No info	No info
Elsaify *et al*. (2006) [22]	British	18	F	Sporadic	4 cm	No	Wide local excision followed by RT	36	No	No
Thanapaisal *et al*. (2006) [17]	Thai	19	F	Sporadic	7 cm	No	Simple mastectomy followed by RT	No info	No info	No info
Kim *et al*. (2006) [16]	Korean	33	F	NF1	(Further information from this article could not be accessed in the scope of our search)					
Dhingra *et al*. (2007) [9]	Indian	38	F	Sporadic	3 cm	No	Lumpectomy	No info	No info	No info
Woo *et al*. (2007) [19]	Korean	56	F	Sporadic	29 cm	No	Modified radical mastectomy with axillary dissection	6	No	No
Wang *et al*. (2009) [21]	Chinese	62	M	Sporadic	2.6 cm	No	Simple mastectomy followed by RT	No info	No info	No info
Yi *et al*. (2009) [5]	Korean	59	F	Sporadic	2.5 cm	No	Wide local excision followed by RT	12	No	No
Akhator *et al*. (2010) [20]	Nigerian	41	F	NF1	10 cm	No	Modified radical mastectomy with axillary dissection	7	Yes (after 9 months)	No
Chalkoo *et al*. (2011) [10]	Indian	60	F	Sporadic	26 cm	No	Simple mastectomy with axillary clearance	1 2	No	No
Manimozhi *et al*. (2015) [18]	Indian	40	F	No info	No info	No	No info	No info	No info	No info

*Abbreviations*: *F* female, *info* information, *LN* lymph node, *M* male, *NF1* neurofibromatosis1, *RT* radiation therapy

Radiological findings were unhelpful in our case due to the fact that no imaging was done before the first surgery. Also, it is certain that a radiological diagnosis of MPNST of the breast is difficult because it shares imaging findings with other neurogenic tumors [19]. By mammography, a well-defined or poorly defined dense nodule with or without calcifications can be seen [26, 30]. Computed tomography (CT) or magnetic resonance imaging (MRI) has little value but some findings should raise suspicion of MPNST such as a large tumor (>5 cm) with heterogeneity, ill-defined margins, invasion of fat planes, and perilesional edema [6, 19, 31]. CT findings of extensive central necrosis and an aggressively growing tumor at the mass periphery could also indicate a diagnosis of MPNST of the breast [19]. Fluorodeoxyglucose-positron emission tomography (FDG-PET), however, has demonstrated reliability in differentiating between benign neurofibromas and MPNST in patients with NF1 in one study [23]; its high degree of accuracy is yet to be proved in other series [23]. In our case, only an ultrasound was done when disease recurred after the first surgery, and it showed five hypoechoic lesions of variable sizes (largest one measuring 3.2×2.6 cm) and a 2.8 cm lymph node at the ipsilateral axilla. Because imaging techniques usually fail to produce characteristic findings, a biopsy is essential to diagnose primary MPNST of the breast [19].

On gross examination, MPNSTs, in most cases, are large, fleshy, often necrotic neoplasms averaging more than 5 cm in diameter [32]. These are fusiform to globular in shape, and may be “white and firm” or “yellow and soft”, depending on the absence or presence of necrosis [25]. Tumors are usually well circumscribed but are not truly encapsulated [25]. The tumor in our case, at first surgery, was composed of multiple nodular pieces of tissue; the larger piece measured 11×11×6 cm. The tumor was mostly solid with cleft-like spaces and gray brown colored. During the fourth surgery, the specimen showed solid gray-white fleshy growth measuring approximately 5×3 cm. Thus macroscopically our case shared a similarity with MPNST as well as with malignant phylloides.

Histological features of MPNST are rather nonspecific. In general, tumors are composed of monotonous spindle cells arranged in intersecting fascicles [23]. In our case, microscopic examination showed that the tumor was composed of predominantly atypical spindle cells with variable cellularity. This is consistent with a spindle cell neoplasm but this spindled type must be carefully distinguished from other spindle cell soft tissue tumors, including malignant phylloides, fibrosarcoma, and leiomyosarcoma [5, 21]. Malignant phylloides shows features reminiscent of phylloides tumor, with compressed epithelium-lined leaf-like spaces [9]. However, microscopic differentiation of malignant phylloides from MPNST can sometimes be difficult. Because of the size and the resemblance to other histological features a diagnosis of malignant phylloides was twice entertained initially in our case. Akhator *et al*. also described the same mistake where a MPNST of the breast was first misdiagnosed as malignant phylloides [20]. Therefore, IHC should have been undertaken to prevent misinterpretation. On morphologic examination, MPNST closely resembles fibrosarcoma [21]. The appearance of MPNST as spindle cells arranged in dense cellular areas interspersed with hypocellular myxoid areas and wavy nuclear contours of its cells should help exclude fibrosarcoma [9]. Our case was also misdiagnosed as fibrosarcoma after the third surgery, which should have been confirmed by immunohistochemical marker. Wang *et al*. have reinforced the necessity of IHC in such situations as it was possible to exclude fibrosarcoma from MPNST of the breast by immunoreactions in their case [21]. In cases of leiomyosarcoma, spindle cells have a more distinct eosinophilic cytoplasm and a blunted nucleus which are less prominent in MPNST, helping diagnosis in this circumstance [9]. A marked raised tumor cellularity, pleomorphism, and mitotic activity and a more organized cellular growth pattern with less extracellular matrix materials distinguish MPNST from otherwise typical neurofibromas [23, 33]. Atypical neurofibromas show increased nuclear pleomorphism without mitotic activity or cellularity [23]. Invasion of surrounding tissues by tumor cells, vascular invasion, and necrosis are other pathological criteria of malignancy [22]. Variable cellularities, presence of necrosis, moderate to marked pleomorphism, and mitotic activity in our case indicated MPNST. In fact, the histological spectrum of MPNST is broad and the diagnosis relies on a combination of some microscopic features, none of which, by themselves, are diagnostic [25].

Because of uncertainty in histological diagnoses, IHC is essential for the definitive diagnosis of MPNST of the breast. Antigens like S100 protein, Leu-7, and myelin basic protein can be used to identify nerve sheath differentiation [22]. According to Farid *et al*., S100 protein is weakly and patchily present in less than 50% of cases, and strong diffuse staining of it nearly always excludes MPNST [23]. The other two antigens show immunoreactivity in approximately half of the tumors [34]. The tumor cells in our case were weakly positive for S100 but negative for CD10, confirming the diagnosis of MPNST at the end of the diagnostic chapter of the clinical scenario.

Because of the rarity, there is no definitive guideline for the management of MPNST of the breast. Successful treatment depends on complete surgical resection [22]. Adjuvant treatment is indicated after wide excision unless the tumor is superficial, small, and low grade [5]. Histologically positive margins or incomplete excision should not be left without further surgery (or adjuvant therapy if surgery is not feasible) [5]. Dissection of axillary tail is not the protocol for patients with clinically negative axilla as the mode of dissemination, like other sarcomas, is primarily hematogenous [5]. Radiotherapy achieves local control and may delay the onset of recurrence but has little effect on long-term survival [5]. Elsaify *et al*. argued the necessity of radiotherapy for high grade tumors, unless margins are very wide, and for intermediate grade tumors with close or positive histologic margins [22]. Chemotherapy has an impact on treating metastatic disease and downstaging unresectable primaries as neoadjuvant setting [5]. The optimum targeted therapy for MPNST is yet to be discovered [23, 25]. Robertson *et al*. reported a 17% response rate with imatinib [35]. Tipifarnib and sorafenib were studied but failed to produce an objective response [36–38]. Trials are going on for bevacizumab, everolimus, nilotinib, sunitinib, and selumetinib [23]. Our case was a high grade tumor with a surgically positive deep margin. Surgery was done several times because of residual lesions. Finally, the disease was controlled locally by the administration of injectable chemotherapy with “IVA” regimen (ifosfamide, vincristine and actinomycin) given 3 weekly for a total of six cycles. Then we treated our patient with three-dimensional conformal radiation therapy with a total dose of 50 Gy given in 25 fractions using a 6 MV linear accelerator followed by 10 Gy in 5 fractions of boost using 9 MeV electron energy.

In general, MPNSTs behave aggressively, and the rate of local recurrence and distant metastases is high. Adverse prognostic factors include tumor larger than 5 cm, presence of von Recklinghausen’s disease, tumor grade, extent of resection, truncal location, and heterologous rhabdomyoblastic differentiation [23, 25]. However, no report is available on median survival or prognosis of MPNST of the breast in the literature [10]. Our patient has not experienced local and metastatic recurrence since completion of all adjuvant treatment after surgery; from the time of first surgery to date she has been surviving well for more than 3 years.

## Conclusions

The unusual primary MPNST of the breast is often unsuspected and the diagnosis may be missed unless clinicians are aware of its signs and symptoms and confounding histopathology. Suspicion of MPNST should be raised in patients with stigmata of NF1 with a breast lump. Special attention should also be paid when a patient without NF1 presents with a rapidly growing painless tumor in and around nerve tissue. A definitive therapeutic protocol needs to be charted but we believe that it can be managed by total mastectomy followed by adjuvant chemotherapy and radiotherapy. Long-term careful follow-up is required in order to generate an optimum treatment strategy.

## Abbreviations

CT: Computed tomography
FDG-PET: Fluorodeoxyglucose-positron emission tomography
HPF: High-power field
IHC: Immunohistochemistry
MPNST: Malignant peripheral nerve sheath tumor
NF1: Neurofibromatosis type 1
VRN: Von Recklinghausen’s neurofibromatosis
